# Women in agriculture: Four myths

**DOI:** 10.1016/j.gfs.2017.10.001

**Published:** 2018-03

**Authors:** Cheryl Doss, Ruth Meinzen-Dick, Agnes Quisumbing, Sophie Theis

**Affiliations:** a International Food Policy Research Institute, IFPRI, 2033 K St Nw, 20036 Washington, DC, United States; b Oxford Department of International Development, University of Oxford, 3 Mansfield Drive, Oxford, United Kingdom

## Abstract

Sustainable Development Goal 5 (SDG) on gender equality and women’s rights and at least 11 of the 17 SDGs require indicators related to gender dynamics. Despite the need for reliable indicators, stylized facts on women, agriculture, and the environment persist. This paper analyzes four gender myths: 1) 70% of the world’s poor are women; 2) Women produce 60 to 80% of the world’s food; 3) Women own 1% of the world’s land; and 4) Women are better stewards of the environment. After reviewing the conceptual and empirical literature, the paper presents the kernel of truth underlying each myth, questions its underlying assumptions and implications, and examines how it hinders us from developing effective food security policies.

## Highlights

• Stylized facts on women, agriculture, and the environment continue to circulate.

• Four myths are inspected against the conceptual and empirical literature.

• These myths hinder the development of effective food security policies.

## 1 Introduction

As the global community mobilizes in support of Sustainable Development Goal (SDG) 5 on gender equality and women's rights, at least 11 of the 17 SDGs require indicators related to gender dynamics. Goal 2, ending world hunger, explicitly mentions addressing the constraints for women small-scale food producers and the nutritional needs of women and adolescent girls. This has contributed to a growing demand for nuanced and accurate data on women's contributions to food security. Despite this emerging global movement for reliable indicators, well-intentioned but statistically unfounded stylized facts on women, agriculture, and the environment continue to circulate. This paper inspects four pervasive gender myths: 1) Women account for 70% of the world's poor; 2) Women produce 60–80% of the world's food; 3) Women own 1% of the world's land; and 4) Women are better stewards of the environment.

These claims are myths. Like all myths, they embody an important truth, in this case that women control fewer resources than those required to fulfill their responsibilities to ensure food and nutrition security for themselves and their families. However, none of these myths are based on sound empirical evidence. While intended to highlight rural women's contributions to food security and natural resource management despite inequality and discrimination, these stylized facts promote stereotypes of women as either victims or saviors; treat women as a monolithic group; ignore the role of men, communities, and institutions; and provide a simplistic and even misleading basis for the design, implementation, and evaluation of policies and programs to promote food security and advance gender equality.

These stylized facts give the impression that they are based on data that are conceptually sound, adequately measured, and statistically representative, when the reality is the reverse. Not only are the underlying data not available, but it is also unclear what data would be needed to support these claims, because the concepts behind the statements are not straightforward. To develop effective policies to promote food security, it is necessary to have appropriate data on women's and men's roles in food production and natural resource management and the gendered constraints that they face. By evaluating the data and assumptions behind these myths, we contribute to both the academic and policy conversations on gender and rural development, making the case for collecting and using better data to capture the variation—over space and time—in the roles and status of women.

## 2 Myth 1: 70% of the world's poor are women

One of the most enduring myths about gender is that 70% of the world's poor are women (UNDP, 1995). Although it is well-documented that women (and girls) worldwide are disadvantaged in terms of schooling, command fewer resources such as land and assets, have earnings and productivity gaps relative to men, and are disadvantaged in terms of voice in their households and society (World Bank, 2012, Food and Agriculture Organization, 2011), the assertion that women comprise 70% of the world's poor has been challenged as far back as the late 1990s.^1^ One needs to question the data on which this myth is based. The most commonly used poverty measures are calculated from income and expenditure data. Incomes and expenditures are flow variables, measured at a point in time, and thereby provide only a snapshot of poverty levels. In contrast, assets are accumulated over time, provide a more holistic picture, and give a better capacity of people to manage their vulnerability to poverty (Deere et al., 2012). Income, expenditure, and asset data are usually collected at the household level, rather than the individual level.

With household level data, there are two options, both unsatisfactory, for calculating gendered poverty rates. The first is to use the sex of the household head and compare male and female headed households. But this ignores women living in male headed households and men living in female headed households. The second option is to allocate household income, expenditures, or assets across household members. To impute consumption expenditures of individuals, it is necessary to make assumptions about the distribution of consumption expenditures within the household, which are implicit in the use of per capita or per adult equivalent measures (Quisumbing et al., 2001a). The use of per capita measures assumes that all members of a household benefit equally from all the inputs received by a household (Alvarado Merino and Lara, 2016); using adult-equivalent measures adjusts for age and sex composition, but still involves assumptions regarding the distribution of resources within a household.

In contrast, a gendered analysis would calculate poverty by using data on the income, consumption, or assets of individuals, rather than households. (Deere et al., 2012).^2^ The main justification for this myth is the alleged predominance of poor, female-headed households, which supposedly contain significantly more female than male members (Marcoux, 1998). But it is not based on individual level data or analysis.

In addition to the flaws of the data on which the myth is based, the myth itself has demographically implausible implications. This assertion implies that men and children make up only 30% of the world's poor, vastly underestimating the number of children in poverty. While “female” includes girls and women, “women” as a demographic category excludes girls (female infants, children, and adolescents), ignoring the different experiences through the lifecycle. Even if this myth were taken to refer to women *and* girls, the demographic implausibility of this assertion was challenged by Marcoux (1998), who pointed out that, if women accounted for 70% of the world's poor, the global population of the poor in the 1990s would comprise 900 million women and girls and 400 million men and boys, or an excess of about 500 million female poor.

There is some evidence that a larger proportion of female-headed households than male-headed households have incomes (or consumption expenditures) below the poverty line. An early review by Buvinic and Gupta (1997), for example, found that 62% of 61 studies that examined the relationship between headship and poverty concluded that woman-headed households are overrepresented among the poor. However, studies that compare poverty incidence based on headship do not tell us who is living in poverty. Because female-headed households account for a much smaller proportion of the population than male headed households, and female-headed households also tend to be smaller households, there are many more women in absolute terms living in male-headed households than there are women living in female-headed households.

Why does debunking this myth matter for food security? Aside from casting women as victims, rather than as contributors to food security, the focus both on women as disproportionately poor and on female-headed households as more vulnerable to poverty can distort the design and implementation of programs and policies. First, this view disregards the heterogeneity among women: there are wealthy women as well as poor women, and characteristics other than gender may be more important for program design and targeting. Second, the focus on female headship may mask important differences among female-headed households (Chant, 2008). For example, female heads of households who receive remittances from a migrant husband, maintain social connections to the husband's family, and expect to have their husband return face fundamentally different opportunities and challenges than a widowed female household head.

The challenge of identifying the poor—both women and men—continues to be important for effective food security and anti-poverty programs. We see three challenges for research and practice. First, the discussion above has all focused on monetary indicators (income, consumption) of poverty and do not capture non-monetary aspects of well-being. Differences in such non-monetary measures of wellbeing as power, nutrition, health, and time allocation may be more important indicators of differences in well-being along gender lines. Some social indicators, notably adult and infant mortality rates, may differ more widely across males and females (Sen, 1998).

As Agbodji et al. (2013) state, poverty measures based on income or consumption remain critically important, but they are insufficient to capture the multidimensional aspects of poverty, especially in poor countries. The Oxford Poverty and Human Development Initiative has developed a cross-national methodology for assessing well-being using the Multidimensional Poverty Index, based on additive and decomposable Alkire and Foster (2011). Agbodji et al. (2013) use nationally representative household surveys from Burkina Faso and Togo to examine inter-country differences in gender inequality. The dimensions of wellbeing considered include some shared by household members, including housing, basic utilities, and assets, and individual-specific aspects, such as education, employment and access to credit. They find both disparities between women and men in terms of multidimensional poverty and sources of inequality that vary across countries and regions. Inequalities in education and employment largely explain gender inequality in Burkina Faso, while those in assets, access to credit and employment are the main sources in Togo.

Second, we need to pay more attention to the measurement of individual incomes, consumption, and assets. The Gender Asset Gap project, the Gender, Agriculture, and Asset Project (GAAP), the LSMS-ISA surveys, Women's Empowerment in Agriculture Index (WEAI) and the Evidence and Data for Gender Equality (EDGE) project are examples of a few efforts to capture information on use, ownership, and control of assets both individually and jointly.^3^

Finally, better measurement and identification of the areas where deprivations are greatest for both men and women should be used to guide the design and implementation of programs that aim to improve food security for the poor.

## 3 Myth 2: Women produce 60–80% of the food

The second myth is that women are the primary food producers in the world. Variations on this claim that women produce 60–80% of food are common. This claim is often made to demonstrate the importance of women's role in agriculture and thus the need to direct policies towards women farmers.

It is well documented that women farmers have less access to land, information, capital and credit, and other inputs than men farmers (see FAO, 2011). In addition, throughout the world, women are the ones primarily responsible for much of the work within the home, including food preparation, cleaning, laundry, and childcare. Thus, if women really were producing most of the world's food, they would be miracle workers.^4^

The kernel of truth in this myth is that women are important for food security, especially within their households. Women's kitchen gardens or homestead plots are often not counted as agriculture, but play an important role, especially in providing dietary diversity. In some contexts, women also grow a large share of the staple cereal or root crops that are consumed by the household. Thus, increasing women's agricultural productivity in these areas can be important for ensuring adequate supplies of macro- and micronutrients for their households. In addition, women contribute to marketed agricultural products through their labor, even if the food produced would not necessarily be attributed to them. This is especially true when they are working as contributing family members or as wage workers on others’ fields.

The first challenge with this claim is how we would attribute a share of the food that is produced to women. While in some cases, women, especially female household heads, may own the land, provide the labor and keep the revenues themselves, most smallholder production relies on the labor of both men and women, making it difficult to allocate the output between them. Other inputs are also required, including management skills, purchased inputs, or extension services, and these would also need to be allocated to individuals within the household in order to apportion the output across people.

Even if we simply focus on labor inputs, it is hard to substantiate the claim that women provide 60–80% of the labor input into food production. FAO reports that 42.2% of the agricultural labor force is women, but even for Africa, the share is less than 50% and it is only 16% for Latin America and the Caribbean (FAO, 2011). An analysis using nationally representative data for six African countries, finds that women provide 24% (Niger) to just over 50% of the labor (Malawi, Tanzania and Uganda) to household crop production (Palacios-Lopez et al., 2016). Labor for livestock production is not included in their study.

The data may underestimate women's involvement in agriculture. The FAO numbers on women's participation in the agricultural labor force may miss women's work. Social norms often result in women saying that they work in the home, even when they are heavily involved in agriculture (see Deere 2005 on Latin America). Furthermore, work in kitchen gardens or tending small livestock or poultry is often not considered agricultural work. Finally, turning agricultural products into food on the table is done primarily by women, although processing and cooking are not counted as agricultural labor.

Better data on women's and men's labor in agriculture and household production are critical for designing policies to promote food security. When new opportunities arise, through changes in markets or technologies, the social norms and traditional patterns of labor will shape who is able to take advantage of them. In particular, women's labor burdens in household work and food production may limit their ability to take advantage of these opportunities.

But simply having data on women's labor in agriculture does not tell us how to increase food security or strengthen rural livelihoods. Even if we knew how much of the world's food was produced by women, how would that inform policy? Instead, to increase food security, we need to understand the gendered constraints faced by both women and men and work to lessen their impacts.

Rather than trying to identify women's independent contributions to agriculture, we need to recognize that agriculture is important for rural women, strengthen women's access to the resources needed for productive agriculture, and reduce the time and energy burdens of household work including food processing and preparation.

## 4 Myth 3: Women own 1–2% of the land

A third myth is that women own 1% or 2% of the world's land – although sometimes the claim is made about property rather than specifically about land. This myth is often linked to issues of food security; women are extensively involved in food production, but are limited because they rarely own the land that they farm.

The myth embodies the truth that both the legal systems and patriarchal gender norms may prohibit or make it difficult for women to acquire and retain land. In addition, almost all inheritance systems disadvantage women in terms of inheritance, and when women legally inherit, they often face strong social pressure to relinquish their inheritance.

When trying to establish the numbers behind this myth, two challenges immediately emerge. The first is what we mean by ownership. If we mean formal documented ownership with the person's name on the title, it might be the case that women only own 1–2% of the land. But men would also own only a small fraction. Much agricultural land is under some form of customary tenure without titles, and where there are titles, often they are not updated to reflect current owners. In some countries, the state retains ownership and men and women only have use rights. If we are interested in who has secure tenure to the land, there is no data at all, let alone sex-disaggregated data. The concepts of owners and managers (or holders) are often used interchangeably in discussions about women's land rights.^5^ In some instance, the person with the rights to the output from the land is considered the owner (or economic owner). In addition, it is not clear how we would consider land that is common property in this framework.

The second challenge is how to handle land that is jointly owned by a man and a woman. If we simply identify the share of land that has a woman as an owner, that might understate the gender gap, since men would also own much of that same land. The best approach is to identify how much land is owned by women, men, and jointly by men and women, but it is harder to use this as a headline statistic.

The sex-disaggregated data on land ownership is still limited, but in Sub-Saharan Africa and Asia, nationally representative data show that women own substantially less land (Doss et al., 2015, Kieran et al., 2015). Yet, in all cases more than 2% is owned by women (see Table 1).

**Table 1:** Share of Household Agricultural Land area held by women, men and jointly by both.

Country (date)	Definition of ownership	Women	Men	Joint
Ethiopia (2011–12)	(registered)	15%	45%	39%
Malawi (2010–11)	(owned)	40%	42%	18%
Niger (2011)	(owned)	9%	62%	29%
Nigeria (2010)	(Right to sell/use as collateral)	4%	87%	9%
Tanzania (2010–11)	(owned)	16%	44%	39%
Uganda (2009–10)	(owned)	18%	34%	48%
Bangladesh (2011–12)	(documented)	10%	88%	2%
Timor-Lest (2007)	(land managers)	88%	12%	n/a
Tajikistan (2007)	(owner)	14%	86%	n/a
Vietnam (2004)	(owner)	72%	15%	13%

The proportion of land that is owned jointly by men and women, typically spouses, ranges from 2% to 48% of household agricultural land. This suggests that in many contexts we miss a key part of the story if we do not consider joint ownership. However, it should not be assumed that joint ownership necessarily provides equal rights over the land; men often have more rights over the land than their wives (Doss et al., 2013, Jacobs and Kes, 2014)

Similar patterns hold when other measures of land rights are used, such as management, or control over output (For Africa, see Slavchevska et al., 2016). The stronger the land rights, the less likely women are to have them.

To ensure food security, it is critical for farmers, both men and women, to have secure tenure to the land that they farm. While both men and women face the risk of losing land due to takeovers from powerful elites and governments or due to a shock that forces them to sell their land, women face an additional layer of risk. Women are often vulnerable to loss of land when their household structure changes, particularly if their husband dies or leaves; in such cases the community or husband's family may take over the land.

Ensuring women's rights to land requires work in two dimensions. First, both land law and family law must protect women's rights. Family law includes both inheritance law and marital property law. Ensuring that women inherit, both from their natal family and from their husband, can further women's land ownership. In addition, the laws regarding property within marriage – whether property acquired during marriage is legally treated as jointly owned (community property) or whether marriage does not confer property rights (separation of property) impacts the extent to which women are owners of land (Deere and Doss, 2006). In contexts of legal pluralism, where both statutory and customary law are both recognized, it is more challenging to ensure that women's land rights are enforced.

The processes of formalizing land rights, through titling or certification, has the potential to either strengthen or weaken women's land rights. Recent efforts to promote women's ownership have had some positive impacts, in contrast to earlier land formalization programs which frequently consolidated land rights in the control of the male household head. Evidence suggests that Rwanda's recent land regularization program improved land access for legally married women and increased investment in soil conservation measures, especially among female-headed households (Ali et al., 2014). The certification process in Ethiopia made an effort to include women's names on the documents; one result is that women heading their own households were more likely to rent out the land that they were not farming, increasing their household welfare (Holden et al., 2011). In Madagascar, where there was little effort to include women in the titling process, relatively few women gained legal rights through this process (Widman, 2014).

Second, women must be aware of their rights, have the ability to enforce them, and be able to challenge social norms that limit women's rights to land. In Ethiopia, Kumar and Quisumbing (2014) found that women were less likely than men to be aware of or to participate in meetings regarding the community-based land registration process that strengthened tenure security. Increasing awareness may involve programs such as mobilizing community workers as paralegals in legal literacy programs (Mueller et al., 2015a, Mueller et al., 2015b).

A major problem with the myth that women own only 1–2% of the land is that it misrepresents the situation on the ground and masks the diversity of tenure situations. The proposed solutions tend to be programs to title land in women's names, when what is needed is more complex. Better data availability on land ownership and land rights, disaggregated by sex, will provide the means to monitor changes over time.

## 5 Myth 4: Women are intrinsically better stewards of the environment

The fourth myth we address does not contain a misleading statistic, but is nonetheless a widely held misperception in the “women and development” domain. Like the other myths, there is a kernel of truth: because of women's traditional roles gathering firewood, collecting water, and managing agriculture, they are greatly affected by natural resource depletion, and therefore have incentives to conserve resources. This view has been reinforced by well-publicized women-led movements for forest protection, such as the Chipko Movement in India and the Green Belt Movement in Kenya (Agarwal, 1992, Mathaai, 2003). Along with protecting the environment, the myth also suggests that women will provide healthy, sustainably grown food to feed their families and communities. Women often have specialized knowledge of certain resources, like medical plants or landraces of crops, and if women are responsible for selecting seeds, they may protect biodiversity. This myth has been useful in drawing attention to that knowledge, which is too often overlooked by external projects that tend to meet primarily with men.

The first major problem with this myth is that it relies on a selective reading of the evidence: the empirical evidence is more mixed (see Meinzen-Dick et al., 2014). Within the forestry sector, for example, Agarwal, 2009, Agarwal, 2010 study of forest user groups in India and Nepal found a positive correlation between the proportion of women on the executive committee and improved forest governance and resource sustainability. On the other hand, a study of forest user groups in Kenya, Uganda, Mexico and Bolivia based on comparative analysis of International Forestry Resources and Institutions (IFRI) data found that female-dominated groups were less likely to adopt new technologies and resource monitoring practices that are associated with improved sustainability, which they attribute to gender biases in technology access, labor constraints, and limitation to women's sanctioning authority (Mwangi et al., 2011, Sun et al., 2011). In Indonesia, Villamor et al. (2013) found that women were more likely to accept hypothetical offers of conversion of forests to oil palm and monoculture rubber plantations, while men expressed stronger conservation beliefs, which may be due to more interaction with conservation agencies.

These findings illustrate a second major problem with this myth: it treats women as a homogenous group and simplifies the relationship between women and nature (Leach, 2007, Goebel, 2003). Using household-level Poverty Environment Network data from 24 countries in Asia, Africa, and Latin America, Sunderland et al. (2014) find that, while there are gendered roles in forest use, men play a larger role than is often assumed (including collecting firewood, often assumed to be women's domain), and that this varies considerably across sites. Women's actions may be motivated more by the material realities of their situation – such as limitations in other resources, a desire to keep their own work burdens from increasing, or a way to guarantee old age support in communities where women do not control resources – than by an inherent connection to nature (Agarwal, 1992, Agarwal, 1997).

Rather than assuming some form of essential conservation orientation of women, recognizing the variability helps to identify other factors that influence conservation, including tenure security, access to information, and complementary resources (such as cash, labor, or force) needed to protect and conserve resources needed for long-term food security. Secure land tenure provides both the incentive and the authority to invest in the resource base, but as noted above, women tend to have less secure tenure than men. In Ghana, Goldstein and Udry (2008) found that women with less secure tenure are less like to leave land fallow to restore soil fertility; conversely, Quisumbing et al. (2001b) found that women with more secure tenure are more likely to plant trees than those who lack tenure security. In Ethiopia, women plot managers with more secure tenure are more likely to plant trees and adopt climate-smart agricultural practices (Quisumbing and Kumar, 2014). Access to information influences uptake of conservation practices, but numerous studies^6^ note that because women have limited access to information due to constraints on mobility, group participation, literacy, or lack of social networks, they are less likely to adopt conservation practices. In a study of conservation practices in Kenya, Bernier et al. (2015) found significant gender gaps in awareness of climate-smart agricultural practices, such as composting, but among those who *are* aware, gender gaps in adoption of composting are narrow – and even reversed. Other complementary resources, including cash or labor, are also needed to adopt many resource-conserving practices.

Finally, this myth can lead to ineffective policies and programs. Targeting women in environmental or climate-smart agriculture projects can lead to increases in their workload (Jackson, 1993, Nightingale, 2006, Torri, 2010). It also ignores the potential and actual complementarities between men and women in terms of their knowledge and skills. In Bangladesh floodplain fisheries, Sultana and Thompson (2008) found greater rule compliance and lower conflicts when the resource management groups involved both men and women. Mixed groups can make the most of men and women's strengths, but they may be more difficult to organize because of the constraints women often face in participating in the “public space” of resource governance, such as forest or water user associations.

We should neither ignore women entirely, nor expect them to be independent drivers of conservation. Rather, we need to work with both men and women and understand the constraints that each face, as well as the gender roles and dynamics between them. Working toward joint resource management groups where both men and women have voice and leadership and toward ensuring that both men and women have secure tenure, information, and other complementary resources is needed to achieve sustainable food security.

## 6 Conclusion

Why do these gender myths continue to circulate? Each of the myths reflect a simple inequity that people intuitively understand: women do a lot with very little. However, grasping for evidence to substantiate this inequity undercuts efforts to redress it. To brush off these myths as harmless inaccuracies ignores the impact they have on gender and development discourse and policy. First, they skew our mental models of gender and development, negatively influencing the design of effective policies and programs. Second, exaggerating gender disparities destroys credibility, obscures the need for better data, and subsequently holds back progress on gender equality.

Researchers, policymakers, and practitioners all carry preconceived notions of gender relations. By shaping and hardening these preconceptions, myths make it more difficult to see nuance, especially nuance that contradicts the myths. Recognizing variation between and within groups of women, women's strengths as well as limitations, and the roles of men is essential for programming that targets and engages the right people, designs goods and services appropriate to their needs, is adaptive to change, and manages risk to avoid harm and unintended consequences.

Narratives that characterize women as either victims or saviors sideline the need to understand women's preferences and priorities. Victim myths assume it is obvious what women need; savior myths assume women want to and can solve all the problems. Both represent a missed opportunity to learn from women and build on their strengths, networks, and knowledge. They also risk increasing women's already heavy work burden, or missing the complementary resources that are needed for women to effectively contribute to sustainable food security.

A final problem with taking these myths as guidance for program design is that by focusing on the needs of women – independent of their families, communities, and institutions – they are blind to gender relations. Altering power dynamics can lead to conflict and backlash against women, and the focus on women, rather than gender, overlooks the contributions of men and the prevalence of “jointness” and negotiation within a household (Johnson et al., 2016). Rejection of the unitary model of the household should not blind us to the possibility of cooperation and complementarity between genders.

The wide circulation of these myths reveals a widespread belief that gender issues in food security are important. Yet if these issues truly are important, better data is necessary to understand them. Stylized facts and statistics distract from major gaps in data on gender and food security, including women's land tenure, control over assets, and work burden, including domestic responsibilities. The lack of nuanced and comprehensive data hinders understanding of the impact of programs and policies. Without it, we are unable to measure change over time, identify drivers of change, examine heterogeneity of impacts, and learn what works to increase food security and gender equality.

In many cases, we can generate better data than the stylized statistics of the myths, not only to dispel them but also to prevent new ones from being propagated. For example, it is possible to collect sex-disaggregated data or measure individual outcomes within a household with only small additions in cost and time. Systematic experiments can be undertaken to assess different ways of collecting data on men and women. Standards for collecting sex-disaggregated data are being recommended for agricultural research institutions and national statistical systems (Doss and Kieran, 2014), and large-scale food security programs are beginning to collect data on men and women within households (Malapit et al., 2014). The development of larger, internationally comparative data sets under the Gender Asset Gap project, GAAP, LSMS-ISA, WEAI, EDGE, IFRI or PEN initiatives can help to go beyond generalizations to a more nuanced and context-specific understanding of men's and women's roles, resources, and constraints. Making progress on gender equality and food security fundamentally requires a better understanding of women and men's lives. This will not come exclusively from quantitative data. Addressing these important issues related to gender and food security will require both robust statistics and qualitative research to understand what men and women experience, what they do, and what they value.

## Footnotes

1 Chant (2006) traces the origin of this myth to the 1990s, catalyzed by the Fourth United Nations Conference on Women in 1995, and which this figure was asserted, and eliminating the “burden of poverty on women: was adopted as one of the 12 critical areas of the Beijing Platform for Action (Chant, 2006, p. 202).

2 There are, however, practical difficulties to individual-based measures. While some components of income are easy to assign to individuals (for example, wages, salaries, and pensions), there are other income components that are difficult to assign owing to the prevalence of joint production within households, such as agricultural production (Doss, 2014) and family-owned enterprises. Although asset-based approaches are more suitable amenable to assessing individual poverty based on individually-owned assets (Deere et al., 2012), many statistical systems still do not collect sex-disaggregated assets data.

3 See http://genderassetgap.org/, http://gaap.ifpri.info, and https://unstats.un.org/edge/. The LSMS-ISA surveys (http://go.worldbank.org/IU0DQRK6G0), in particular, are now available for a range of countries in Africa. Some of the more recent ones collect sex-disaggregated information on land ownership, land management and control of harvest. In addition, LSMS-ISA surveys collect information on individual incomes and some even collect individual-level information on various assets, though those are often reported by a proxy rather than by the owner/holder himself/herself.

4 See Doss (2014) for a detailed discussion of this myth.

5 FAO data from agricultural surveys and censuses tends to use the concept of a holder, or manager, rather than landowner.

6 See Peterman et al., 2010 for a review.
